# The Liaison between Metabolism and Oxidative Stress in Human Diseases

**DOI:** 10.3390/cells12242823

**Published:** 2023-12-12

**Authors:** Silvia Ravera, Isabella Panfoli

**Affiliations:** 1 Department of Internal Medicine and Medical Specialties, University of Genoa, 16132 Genoa, Italy; silvia.ravera@unige.it; 2 Departiment of Pharmacy (DIFAR), University of Genoa, 16132 Genoaa, Italy

Mitochondria have been the prerequisite to eukaryote complexity since their likely endosymbiotic origin, allowing a remarkable expansion in the number of genes expressed [1]. Mitochondria play a pivotal role in eukaryotic cell bioenergetics as the principal source of ATP. However, metabolic efficiency comes at a cost: catabolism is linked to reactive oxygen species (ROS) production. Mitochondrial oxidative phosphorylation (OxPhos) is the leading ROS producer. Oxidative stress is characterized by an imbalance between the production of ROS and the antioxidant capacity, resulting in cellular oxidative injury and disturbances in cell homeostasis [2]. Mitochondria are also dynamic organelles undergoing fusion and fission, involved in many cellular activities regulation, including cell death, cell proliferation, and mitophagy. The latter is a quality check that can remove damaged mitochondria or reduce the mitochondrial mass, mitigating oxidative stress. In addition, the interaction between the mitochondria network and endoplasmic reticulum (ER) is a new and constantly expanding chapter that is not limited to calcium homeostasis but to the modulation of the cellular energy and redox state [3]. Evidence suggests that cellular redox homeostasis is linked to mitochondrial dynamics in that fusion and fission processes are affected by ROS [2]. A novel aspect of the mitochondrial dynamics may be the transfer of mitochondrial OxPhos machinery, once assembled inside the mitochondria, to other membranes where these are ectopically expressed. In some cells, the OxPhos would also take place in membranous structures other than mitochondria, such as the rod outer segments and platelets [4]. Mitochondrial dysfunction can severely impact the cell, inducing hypometabolism and triggering apoptosis. In recent years, our understanding of the role of mitochondria-related oxidative stress in various diseases, including cancer, diabetes, cardiovascular disease, renal damage, and neurodegenerative disorders, has sensibly improved [5]. A recent report suggests the role of intracellular beta cell oxidative stress in initiating and promoting autoimmunity during T1D type 1 diabetes (T1D). Hyperglycemia can cause oxidative stress, damaging the beta cells and triggering autoimmunity [6]. Evidence shows a relationship between mitochondrial oxidative dysfunction and the progression of kidney injury and disease. The mitochondrial network and its contact with other cellular organelles, such as the endoplasmic reticulum, are oxidative stress risk factors at the basis of kidney disease. Therapies balancing mitochondrial turnover and improving mitochondrial homeostasis have been proposed for renal disease. There is a link between mitochondrial ROS and cardiac disease: mitochondria are abundant in cardiac tissue: smoking, diabetes, high blood pressure, and hypercholesterolemia increasing ROS generation or imbalance in antioxidant defenses are common risk factors for cardiovascular diseases [5]. There is a link between the activation of platelets and endothelial dysfunction: a damaged endothelium during inflammation or hypoxia promotes platelet activation [7]. Platelet metabolism, in turn, may depend both on mitochondrial and ectopically located oxidative phosphorylation. Oxidative stress is involved in cancer pathophysiology: increased ROS production activates pro-tumorigenic signaling, causing genetic instability. In several tumors, the end products of peroxidation are elevated, and antioxidant enzymes are reduced. On the other hand, oxidative stress can also trigger tumor cell death. A recognized commonality between cancer incidence and aging is mitochondrial dysfunction and the consequent oxidative stress [8]. Oxidative stress is linked to the etiology of many neurodegenerative diseases, such as Alzheimer’s disease, amyotrophic lateral sclerosis, multiple sclerosis, and Parkinson’s disease [9]. Mitochondrial dysfunction is commonly observed in various ocular diseases, including age-related macular degeneration (AMD) and diabetic retinopathy (DR). The source of oxidative stress in Parkinson’s disease may be the impairment in complex I of the mitochondrial electron transport chain, contributing to the degeneration of dopaminergic neurons [10].

Another aspect of the role of ROS in disease that requires further investigation is their dual role. There are examples of reciprocal regulation between antioxidant and energy production pathways, demonstrating that ROS playa role in signaling the cellular redox state and also play a physiological role in activating the antioxidant response [10]. There is a focus on the transcription factor called nuclear factor (erythroid-derived 2)-like 2 (Nrf2) that changes the expression of the antioxidant enzymes (superoxide dismutase, catalases, and glutathione peroxidases) [11]. Nrf2 can also modulate the expression of genes that control immune and inflammatory responses, fibrosis, carcinogenesis, and cognitive dysfunction [11].

An inverse relationship exists between plant-based food consumption rich in phytochemicals such as polyphenols and cancer incidence, modulating several oxidative stress-mediated signaling pathways, among which the F_1_F_o_-ATP synthase [12]. Polyphenols can also prevent cardiovascular and neurodegenerative diseases. The Special Issue addressed all the topics mentioned in an attempt to answer the many unanswered questions.

A primary focus of future research should be the identification of the metabolic pathways involved in ROS formation as the prerequisite pivotal to our understanding of the molecular basis of oxidative stress-related diseases and for the design of their novel therapeutic strategies. Mechanistic aspects of the selective impact on high energy-demanding cells that are, however, relatively devoid of mitochondria, such as neurons, are still unclear. For example, mitochondrial diseases, a group of inherited rare disorders characterized by defective oxidative phosphorylation due to mutations in respiratory complex subunits or assembly factors and proteins involved in mitochondrial fusion and fission, interestingly affect primarily the visual system and the central and peripheral nervous system, which are relatively poor in mitochondria. Future studies may provide insight into ROS signaling pathways manipulation, i.e., the complex regulatory axis of redox status playing signaling roles, promoting autophagy during cell starvation or metabolic stress while still fully characterizing the pathways encompassing AMPK, mTOR, and Akt. Focusing on the molecular aspect of the action of phytochemicals can refine our ability to use them as preventative or therapeutic options for the cited diseases.

This Editorial refers to the Special Issue “*Recent Advances in Metabolism and Oxidative Stress in Human Diseases*”. The Special Issue focused on how the efficacy of ATP aerobic production by the mitochondrial OxPhos metabolism can cause the production of reactive oxygen species (ROS), which may lead to the onset of oxidative stress. The latter is emerging as the ultimate cause of multiple diseases. Eleven manuscripts were submitted for consideration for the Special Issue, all of which were subjected to a rigorous review process. In total, nine papers were finally accepted for publication and inclusion in this Special Issue.

Special Issue (six articles, two reviews, and one perspective). The contributions are listed below:

1. Algieri, C.; Bernardini, C.; Oppedisano, F.; La Mantia, D.; Trombetti, F.; Palma, E.; Forni, M.; Mollace, V.; Romeo, G.; Nesci, S. Mitochondria Bioenergetic Functions and Cell Metabolism Are Modulated by the Bergamot Polyphenolic Fraction. *Cells* **2022**, *11*, 1401. https://doi.org/10.3390/cells11091401.
2. Golchert, J.; Staar, D.; Bennewitz, J.; Hartmann, M.; Hoffmann, N.; Ameling, S.; Völker, U.; Peters, J.; Wanka, H. Overexpression of Renin-B Induces Warburg-like Effects That Are Associated with Increased AKT/mTOR Signaling. *Cells* **2022**, *11*, 1459. https://doi.org/10.3390/cells11091459.
3. Bertola, N.; Degan, P.; Cappelli, E.; Ravera, S. Mutated FANCA Gene Role in the Modulation of Energy Metabolism and Mitochondrial Dynamics in Head and Neck Squamous Cell Carcinoma. *Cells* **2022**, *11*, 2353. https://doi.org/10.3390/cells11152353.
4. Bajia, D.; Bottani, E.; Derwich, K. Effects of Noonan Syndrome-Germline Mutations on Mitochondria and Energy Metabolism. *Cells* **2022**, *11*, 3099. https://doi.org/10.3390/cells11193099.
5. Carlini, L.; Tancreda, G.; Iobbi, V.; Caicci, F.; Bruno, S.; Esposito, A.; Calzia, D.; Benini, S.; Bisio, A.; Manni, L.; et al. The Flavone Cirsiliol from Salvia x jamensis Binds the F1 Moiety of ATP Synthase, Modulating Free Radical Production. *Cells* **2022**, *11*, 3169. https://doi.org/10.3390/cells11193169.
6. Gomes, D.; Joubert, A.; Visagie, M. The Biological Relevance of Papaverine in Cancer Cells. *Cells* **2022**, *11*, 3385. https://doi.org/10.3390/cells11213385.
7. Savic Vujovic, K.; Zivkovic, A.; Dozic, I.; Cirkovic, A.; Medic, B.; Srebro, D.; Vuckovic, S.; Milovanovic, J.; Jotic, A. Oxidative Stress and Inflammation Biomarkers in Postoperative Pain Modulation in Surgically Treated Patients with Laryngeal Cancer—Pilot Study. *Cells* **2023**, *12*, 1391. https://doi.org/10.3390/cells12101391.
8. Ravera, S.; Signorello, M.; Panfoli, I. Platelet Metabolic Flexibility: A Matter of Substrate and Location. *Cells* **2023**, *12*, 1802. https://doi.org/10.3390/cells12131802.
9. Ravera, S.; Bertola, N.; Puddu, A.; Bruno, S.; Maggi, D.; Panfoli, I. Crosstalk between the Rod Outer Segments and Retinal Pigmented Epithelium in the Generation of Oxidative Stress in an In Vitro Model. *Cells* **2023**, *12*, 2173. https://doi.org/10.3390/cells12172173.
